# The METTL3/MALAT1/PTBP1/USP8/TAK1 axis promotes pyroptosis and M1 polarization of macrophages and contributes to liver fibrosis

**DOI:** 10.1038/s41420-021-00756-x

**Published:** 2021-11-27

**Authors:** Bo Shu, Ying-Xia Zhou, Hao Li, Rui-Zhi Zhang, Chao He, Xin Yang

**Affiliations:** 1 Department of General Surgery, The Second Xiangya Hospital, Central South University, 410011 Changsha, Hunan Province China; 2 Department of Surgical Operation, The Second Xiangya Hospital, Central South University, 410011 Changsha, Hunan Province China

**Keywords:** Diseases, Gastrointestinal diseases

## Abstract

Pro-inflammatory M1 macrophages, via activating hepatic stellate cells, contribute to liver fibrosis. In this study, we examined the mechanism and the significance of a signaling axis, METTL3/MALAT1/PTBP1/USP8/TAK1, in regulating pyroptosis and M1 polarization of hepatic macrophages. Liver fibrosis model was established in vivo by CCl_4_ treatment; M1 polarization was induced in vitro by treating macrophages with lipopolysaccharide or interferon γ. Expressions of METTL3, MALAT1, PTBP1, USP8, and TAK1 were measured by RT-PCR and/or Western blot in Kupffer cells (KCs) isolated from in vivo model or in vitro activated macrophages. Macrophage phenotypes including inflammation (RT-qPCR analysis of a panel of proinflammatory cytokines and ELISA on productions of interleukin (IL)−1β and IL-18) and pyroptosis (Western blot of NLRP3, Caspase-1, and GSDMD) were investigated. The impact of METTL3 on m^6^A methylation of MALAT1 was examined by methylated RNA immunoprecipitation (RIP), the interaction between PTBP1 and MALAT1 or USP8 mRNA by combining RNA pull-down, RIP, and RNA stability assays, and the crosstalk between USP8 and TAK1 by co-immunoprecipitation and protein degradation assays. Functional significance of individual component of METTL3/MALAT1/PTBP1/USP8/TAK1 axis was assessed by combining gain-of-function and loss-of-function approaches. In KCs isolated from in vivo liver fibrosis model or in vitro M1-polarized macrophages, METTL3 was up-regulated, and sequentially, it increased MALAT1 level via m^6^A methylation, which promoted USP8 mRNA degradation through the interaction with PTBP1. Reduced USP8 expression regulated the ubiquitination and protein stability of TAK1, which promoted pyroptosis and inflammation of macrophages. The signaling cascade METTL3/MALAT1/PTBP1/USP8/TAK1, by essentially stimulating pyroptosis and inflammation of macrophages, aggravates liver fibrosis. Therefore, targeting individual components of this axis may benefit the treatment of liver fibrosis.

## Introduction

Liver fibrosis is a chronic liver injury that may develop from long-term alcohol consumption, infection with hepatitis B or C viruses (HBV or HCV), or metabolic disorders [1, 2]. In China, improvements in people’s lifestyles and the prevalence of HBV and HCV infections are contributing to the increasing morbidity and mortality of liver fibrosis and deteriorating the quality of life for these patients [3]. The activation and the differentiation of hepatic stellate cells (HSCs) into fibrogenic myofibroblasts are a driving force for the pathogenesis of liver fibrosis, which are initiated through the interaction with immune cells such as Kupffer cells (KCs), monocyte-derived macrophages, natural killer cells, B cells, and T cells [4]. The resident KCs and monocyte-derived macrophages are the two major sources of hepatic macrophages and play critical roles in maintaining liver homeostasis and shaping the development of liver diseases [5]. In response to different microenvironmental stimuli, hepatic macrophages may present heterogenous functions ranging from the pro-inflammatory M1 phenotype to the anti-inflammatory M2 phenotype [6]. Specifically, M1 macrophages, characterized by increased expressions and productions of pro-inflammatory cytokines or inflammation-related molecules including interleukin (IL)−1β, IL-6, IL-18, tumor necrosis factor (TNF)-α, monocyte chemoattractant protein-1 (MCP1), lymphocyte antigen 6 complex (Ly6c), and iNOS, promote the activation of HSCs [6, 7]. Therefore, understanding the mechanisms controlling the M1 polarization of hepatic macrophages may help identify strategies that target the activation of HSCs and the development of live fibrosis.

While searching for the mechanisms regulating M1 polarization of hepatic macrophages and thus potentially modulating the progression of liver fibrosis, we came across data suggesting that a signaling axis, m^6^A-catalytic enzyme methyltransferase like 3 (METTL3)/metastasis-associated lung adenocarcinoma transcript 1 (MALAT1; a long non-coding RNA (lncRNA))/polypyrimidine tract-binding protein 1 (PTBP1; an RNA-binding protein)/ubiquitin-specific peptidase 8 (USP8; a deubiquitinating enzyme)/transforming growth factor β-activated kinase 1 (TAK1, a kinse), may promote pyroptosis and M1 polarization of hepatic macrophages. First, there is a reciprocal positive feedback between up-regulated METTL3 and M1 polarization of macrophages [8]. Second, MALAT1 is essential for M1 polarization of macrophages [9], while METTL3 stabilizes MALAT1 via m^6^A methylation [10]. Third, pyroptosis is an inflammatory form of programmed cell death that depends on the cleavage of pro-caspase-1 and gasdermin D (GSDMD) to caspase-1 and GSDMD-N, respectively, and to form cell membrane pores to release inflammatory cytokines, including IL-1β and IL-18 [11]. Increasing evidence suggests that pyroptosis is linked to M1 polarization of macrophages and the development of liver fibrosis [12, 13]. Accordingly, it is reasonable to predict that TAK1, an essential regulator for pyroptosis in macrophages [14], may contribute to M1 polarization of macrophages and liver fibrosis. Considering that MALAT1 stabilizes PTBP1 [15], USP8 deteriorates the stability of TAK1 [16], and our preliminary analysis using bioinformatic tools, StarBase (http://starbase.sysu.edu.cn/) and RNAInter (www.rna-society.org/rnainter/), revealed the potential bindings between PTBP1 and MALAT1 as well as USP8, we hypothesize that METTL3 induces sequential stabilizations of MALAT1 and PTBP1, and the latter promotes the degradation of USP8 mRNA and thus improves the stability of TAK1 protein. Functionally, we expect the METTL3/MALAT1/PTBP1/USP8/TAK1 axis induces pyroptosis and M1 polarization of macrophages. To test this hypothesis, we used liver fibrosis as the disease model and specifically focused on hepatic macrophages in this model.

## Results

### MALAT1 and METTL3 were up-regulated in Kupffer cells (KCs) following the in vivo progression liver fibrosis, and in macrophages activated in vitro with IFN-γ or LPS

**Fig. 1:**
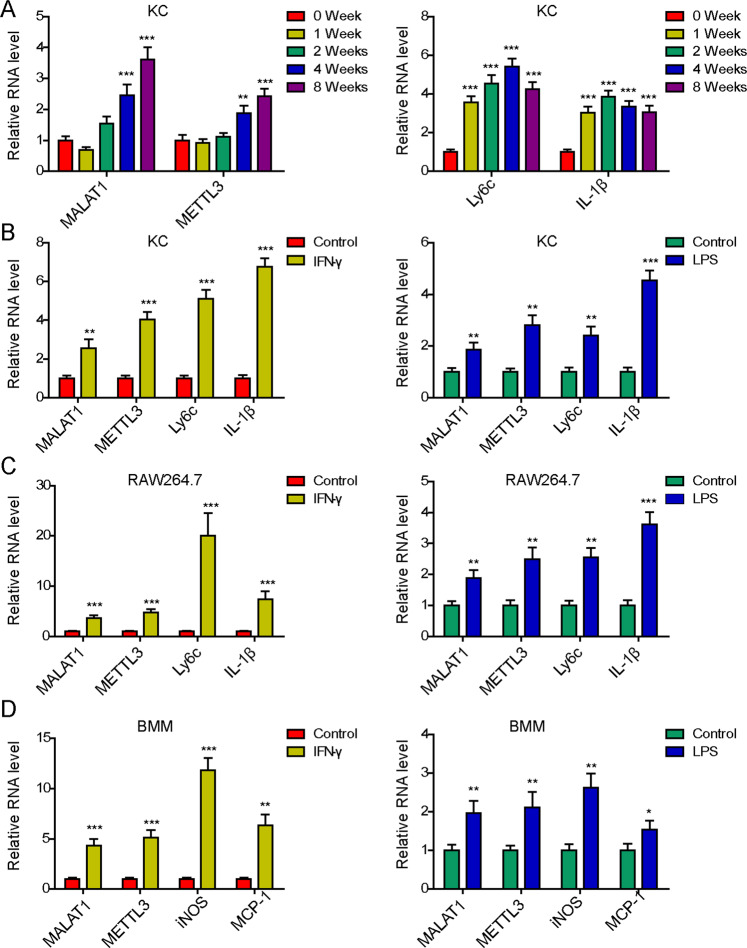
MALAT1 and METTL3 were up-regulated in Kupffer cells (KCs) during liver fibrosis, and in M1 macrophages activated with IFN-γ or LPS. Mice were treated with CCl4 to induce liver fibrosis. **A** Expressions of MALAT1, METTL3, Ly6c, and IL-1β were examined by RT-PCR in KCs isolated from mice treated with CCl4 for 0, 1, 2, 4, and 8 weeks, respectively. KCs (**B**), RAW264.7 (**C**), and BMMs (**D**) were stimulated with either IFN-γ or LPS for 24 h and expressions of indicated genes were measured by RT-PCR. *P < 0.05, **P < 0.01, ***P < 0.001.

To understand the interaction between MALAT1 and METTL3 within macrophages during liver fibrosis, we first established an in vivo CCl_4_-induced liver fibrosis model. Upon injecting mice with CCl_4_ for different time periods, 0, 1, 2, 4, and 8 weeks, we isolated KCs and measured the expressions of MALAT1, METTL3, and two markers for proinflammatory M1 macrophages, Ly6c and IL-1β. As shown in Fig. 1A, MALAT1 and METTL3 expressions presented a time-dependent increase with the progression of CCL4-induced liver fibrosis. The expressions of Ly6c and IL-1β also robustly increased in response to CCl_4_ injection, but the former peaked at week 4 and the latter, week 2.

In addition to examining MALAT1 and METTL3 expressions using the in vivo liver fibrosis model, we also established an in vitro model where macrophages were treated with IFN-γ or LPS to induce M1 polarization, which are well demonstrated to promote the productions of pro-inflammatory cytokines, activate HSCs, and stimulate liver fibrosis [7, 17]. By applying IFN-γ or LPS to KCs isolated from wild-type mice, immortalized macrophage cell line RAW264.7, or bone-marrow-derived macrophages (BMMs), we found that both stimuli successfully induced inflammation in these macrophages, as represented by the up-regulations of Ly6c and IL-1β in KCs (Fig. 1B) and RAW264.7 cells (Fig. 1C), and those of iNOS and MCP-1 in BMMs (Fig. 1D). Concomitantly, we observed the increase of both MALAT1 and METTL3 in these cells, suggesting that the up-regulations of MALAT1 and METTL3 in macrophages were closely associated with the development of liver fibrosis.

### Knocking down MALAT1 inhibited the activation of proinflammatory macrophages and NLRP3 inflammasome-induced pyroptosis during the development of liver fibrosis

**Fig. 2:**
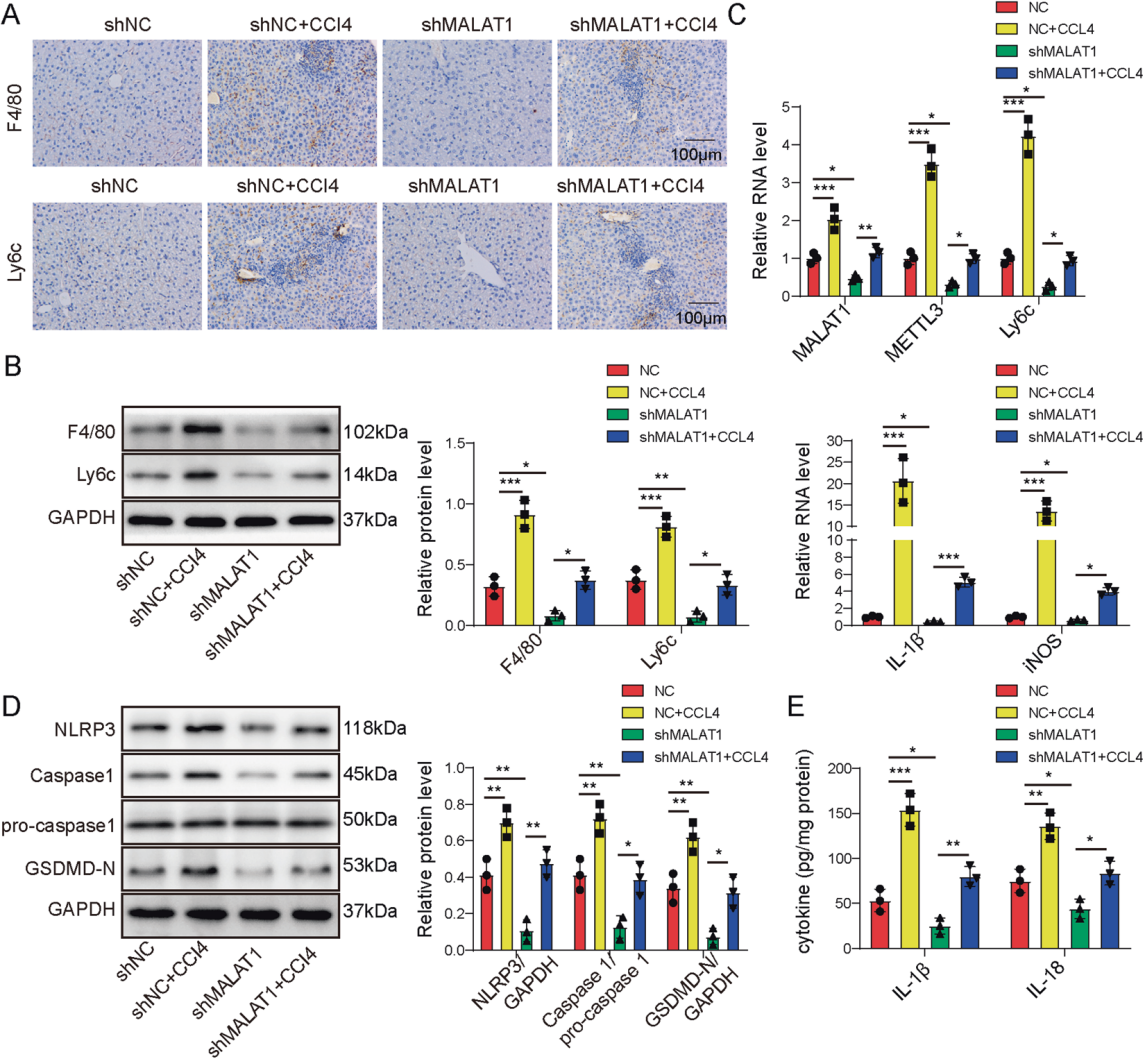
Knocking down MALAT1 inhibited the activation of proinflammatory macrophages and NLRP3 inflammasome-induced pyroptosis. Mice were injected shNC, shMALAT1, CCl4 in combination with shNC (shNC + CCl4), or CCl4 in combination with sh-MALAT1 (shMALAT1 + CCl4) (*n* = 10/group). Expressions of F4/80 and Ly6c proteins were detected by immunohistochemistry (**A**) and by western blot (**B**) in liver tissues from indicated groups. **C** The expressions of MALAT1, F4/80, Ly6c, IL-6, and iNOS were examined by RT-qPCR. **D** The expressions of pyroptosis-related markers, NLRP3, pro-caspase-1, Caspase-1, and GSDMD-N from liver tissues of indicated groups were examined by western blot. **E** The levels of IL-1β and IL-18 in the liver tissues were measured by ELISA from indicated groups. *P < 0.05, **P < 0.01, ***P < 0.001.

To assess the functional significance of MALAT1 in M1 macrophage activation and liver fibrosis, we injected either control shRNA (shNC) or MALAT1-specifid shRNA (shMALAT1) directly into mice before inducing liver fibrosis with CCl_4_. We found that shMALAT1 significantly inhibited the activation of M1 macrophages in response to CCl_4_, as demonstrated by the marked reduction of F4/80^+^ or Ly6c^+^ cells (Fig. 2A) or their expressions in isolated KCs (Fig. 2B, C) in shMALAT1 + CCl_4_ mice than in shNC + CCl_4_ mice, and there was a similar difference between shNC and shMALAT1 mice (Fig. 2A–C). Similar changes were also observed for proinflammatory makers, IL-1β and iNOS in isolated KCs from corresponding mice (Fig. 2C).

Considering the pathogenic importance of NLRP3 inflammasome-induced pyroptosis in liver fibrosis, we measured the levels of key markers for pyroptosis, including NLRP3, pro-caspase-1, cleaved Caspase-1, cleaved GSDMD-N (Fig. 2D), IL-1β, and IL-18 (Fig. 2E). We found that CCl4 by itself robustly increased the levels of NLRP3, Caspase-1, an GSDMD-N, while shMALAT1 potently antagonized the effects of CCl4. All treatments had minimal impacts on the level of pro-caspase-1. Collectively, these data suggest that MALAT1 is essential for activating M1 macrophages and NLRP3 inflammasome-mediated pyroptosis in CCl_4_-induced liver fibrosis.

### Targeting MALAT1 dampened inflammation and NLRP3 inflammasome-induced pyroptosis in M1 macrophages

**Fig. 3:**
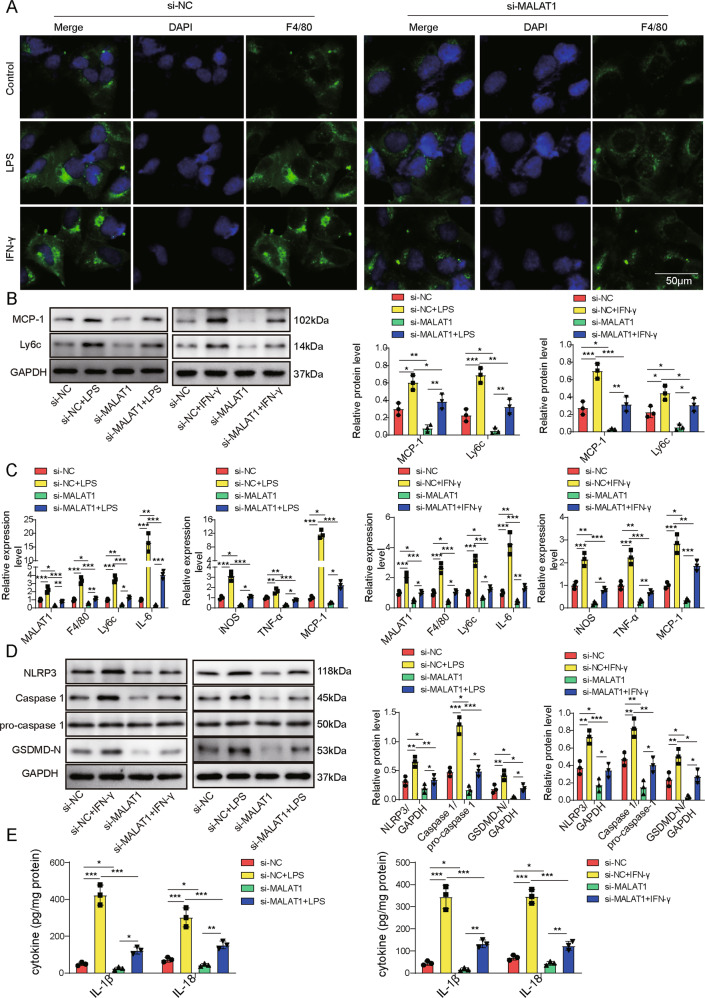
Targeting MALAT1 dampened inflammation and NLRP3 inflammasome-induced pyroptosis in M1 macrophages. Isolated KCs were transfected with siNC or siMALAT1 and treated with PBS (control), LPS, or IFN-γ. **A** F4/80 expression in indicated cells was examined by immunofluorescence (green). Cell nuclei were stained with DAPI (blue). Expressions of F4/80 and Ly6c proteins were detected by western blot (**B**), of MALAT1, F4/80, Ly6c, IL-6, iNOS, TNF-α, and MCP-1 by RT-qPCR (**C**), and of pyroptosis-related markers, NLRP3, pro-caspase-1, Caspase-1, and GSDMD-N by western blot (**D**) from indicated cells. **E** The productions of IL-1β and IL-18 in supernatants of indicated cells were measured by ELISA. *P < 0.05, **P < 0.01, ***P < 0.001.

Next, we examined the impacts of targeting MALAT1 in isolated KCs stressed with IFN-γ or LPS. Either stress significantly up-regulated surface expression of F4/80 on si-NC-transfected KCs but failed to do so on those transfected with si-MALAT1 (Fig. 3A). Meanwhile, we detected no changes in pro-caspase-1 (Fig. 3D), but increased proteins levels of MCP-1, Ly6c (Fig. 3B), NLRP3, cleaved Caspase-1, and GSDMD-N (Fig. 3D), mRNA levels of MALAT1, F4/80, Ly6c, IL-6, iNOS, TNF-α, and MCP-1 (Fig. 3C), and secretions of IL-1β and IL-18 (Fig. 3E) into the culture medium of si-NC cells treated with IFN-γ or LPS, but not in IFN-γ- or LPS-treated si-MALAT1 KCs, suggesting that MALAT1 critically controlled inflammation and pyroptosis of activated macrophages.

### METTL3 increased MALAT1 level through m^6^A modification

**Fig. 4:**
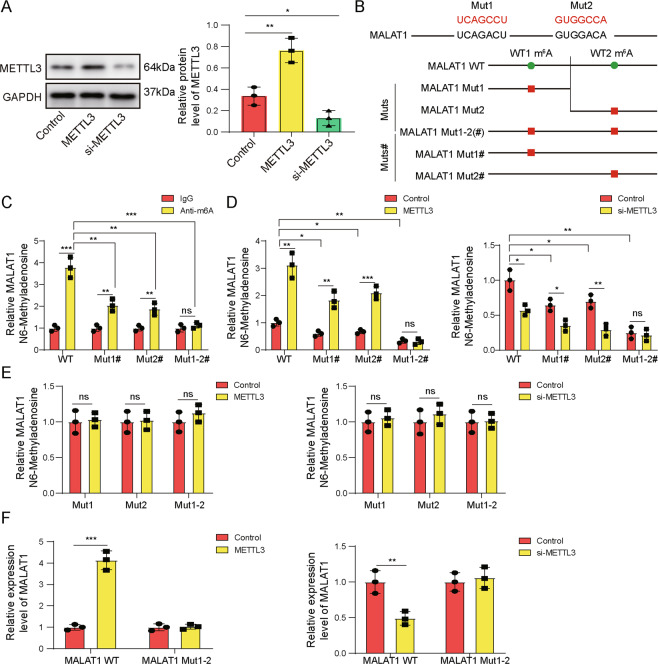
METTL3 increased MALAT1 level through m6A modification. METTL3 was either overexpressed (METTL3) or knocked down (si-METTL3) in isolated KCs. **A** The protein level of METTL3 was examined by western blot in indicated cells. Control, parental KCs. **B** Wildtype and mutated (Mut; red letters) m6A methylation sequences in MALAT1 predicted using m6Avar database and constructs containing different mutations were illustrated. **C** m6A methylation on indicated MALAT1 constructs was examined by m6A mRNA immunoprecipitation assay followed by RT-qPCR. **D**, **E** m6A methylation status on indicated MALAT1 constructs in parental KCs, those overexpressing METTL3, or knocking down METTL3 (si-METTL3) was examined by m6A mRNA immunoprecipitation assay followed by RT-qPCR. **F** METTL3-expressing (left panel) or si-METTL3-expressing (right panel) KCs were transfected with MALAT1-WT or MALAT1-Mut1-2. The expressions of MALAT1 were analyzed by RT-qPCR. *P < 0.05 or **P < 0.01, ***P < 0.001.

An earlier study showed that METTL3 formed complex with YTHDF3 and stabilized MALAT1 through m^6^A modification [10]. The concurrent up-regulation of both MALAT1 and METTL3 in activated macrophages prompt us to examine whether the same mechanism operates in these cells. For this purpose, we transiently overexpressed or knocked down METTL3 with si-METTL3 in isolated KCs (Fig. 4A). Analysis of m^6^Avar database (http://m6avar.renlab.org/) revealed two sequence motifs (#1 and #2) for m^6^A modification in MALAT1, based on which, synonymous mutations (indicated by the red letters; Fig. 4B) were introduced and several mutant sequences were generated: WT containing both wild-type (WT) #1 and WT#2 motifs, Mut1 containing only mutated #1 but not #2 m^6^A sequence motif, Mut2 containing only mutated #2 but not #1 m^6^A sequence motif, Mut1-2(#) containing both mutated #1 and mutated #2 motifs, MALAT1 Mut1# containing mutated #1 and WT #2 motifs, and MALAT1 Mut2# containing WT #1 and mutated #2 motifs (Fig. 4B). Methylated RNA immunoprecipitation (RIP) assay showed that in non-transfected control KCs, m^6^A modification were detected on both sequence motifs and Mut1-2(#) completely abolished m^6^A modification (Fig. 4C). Overexpressing METTL3 in KCs significantly boosted m^6^A modification on both WT motifs, but not on Mut1-2(#), while si-METTL3 potently reduced it (Fig. 4D). In addition, deletion of either sequence motif disrupted the m^6^A modification on the other motif in response to ectopic expressing or knocking down METTL3 (Fig. 4E). Functionally, co-transfecting METTL3 with MALAT1 WT into KCs increased the expression of the latter, but with MALAT Mut1-2 abolished it; co-transfecting si-METTL3 presented the opposite effect on MALAT1 WT but not Mut1-2 expression (Fig. 4F). Collectively, these data suggest that by promoting m^6^A modification, METTL3 increases MALAT1 level.

### MALAT1 directly interacted with PTBP1 and down-regulated USP8

**Fig. 5:**
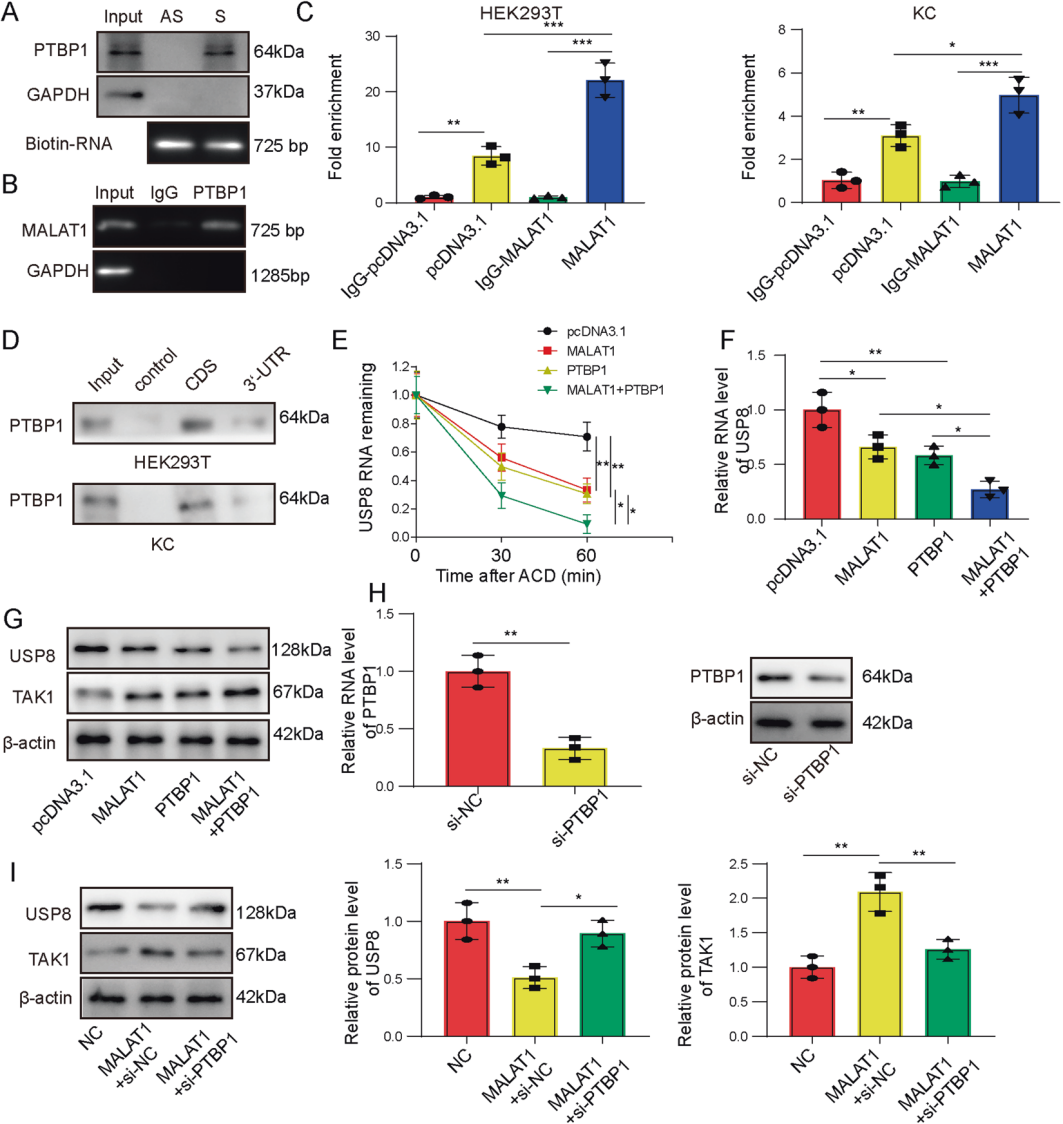
MALAT1 directly interacted with PTBP1 and down-regulated USP8. **A** Biotin-labeled sense (S) and antisense (AS) MALAT1 were synthesized and incubated with KCs lysates to detect the putative interaction with PTBP1. **B** The interaction between endogenous MALAT1 and PTBP1 in KCs cells was examined by RIP assay. **C** The interaction between PTBP1 and USP8 was detected by RIP assay in HEK293T (left panel) or KCs (right panel) overexpressing MALAT1 or not (pcDNA). IgG was used as the negative control. **D** Biotin-labeled CDS and 3’-UTR of USP8 were in vitro synthesized and incubated with HEK293T or KC lysates. Specific binding of PTBP1 on different constructs was detected by western blot. **E** KCs were transfected with pcDNA, MALAT1, PTBP1, or MALAT1 + PTBP1, and treated with actinomycin D (ACD) to block de novo transcription. The mRNA level of USP8 was examined by RT-qPCR at indicated time points after ACD application. **F** The mRNA levels of USP8 were examined by RT-qPCR in indicated KCs. **G** The protein levels of USP8 and TAK1 were examined by western blot in indicated KCs. **H** KCs were transfected with si-NC or si-PTBP1. Expression of PTBP1 was examined by RT-qPCR and western blot. **I** The protein levels of USP8 and TAK1 were examined by western blot in indicated KCs with altered expressions of MALAT1 and PTBP1. *P < 0.05 or **P < 0.01, ***P < 0.001.

To understand the pyroptosis-promoting mechanism of MALAT1 in activated macrophages, we performed bioinformatic analysis using StarBase and RNAInter, and found that PTBP1 may interact with MALAT1 as well as USP8. A recent study also reported that MALAT1 directly interacted with and stabilized PTBP1 [15]. In KC cells, using both RNA pull-down (Fig. 5A) and RIP (Fig. 5B), we detected specific interactions between MALAT1 and PTBP1. RIP assay also revealed the specific binding of PTBP1 on USP8 in both HEK293T and KCs, which was further boosted when overexpressing MALAT1 in these cells (Fig. 5C). Using biotin-labeled RNA probe containing the coding region (CDS) or 3′-UTR of USP8 mRNA, we found that PTBP1 bound to CDS and to a much lesser extent, 3′-UTR in HEK293T cells, but only to CDS in KCs (Fig. 5D). Since PTBP1 is a critical regulator of mRNA stability, we tracked the stability of USP8 mRNA upon treating KCs with actinomycin D, an inhibitor for de novo transcription. We found that overexpressing either MALAT1 or PTBP1 significantly promoted degradation of USP8 mRNA. When both MALAT1 and PTBP1 were overexpressed, the mRNA level was the lowest (Fig. 5E). Consistently, we found that ectopically expressing either MALAT1 or PTBP1 significantly reduced the steady-state mRNA level of USP8 in KCs and the most robust reduction was observed in cells co-transfected with both MALAT1 and PTBP1 (Fig. 5F). This effect also translated to the protein level of USP8 and an opposite effect on TAK1 (Fig. 5G). In addition to the gain-of-function strategy, we examined the effect of targeting PTBP1 with siRNA (si-PTBP1) on USP8 expression. We found that si-PTBP1 was sufficient to antagonize the inhibitory effect of MALAT1 on USP8 (Fig. 5H, I), which resulted in an increase USP8 and a decrease of TAK1 on the protein levels in MALAT1 + si-PTBP1 KCs, when compared to MALAT1 + si-NC cells (Fig. 5I). Overall, these data suggest that MALAT1 reduces USP8 and up-regulates TAK1 levels through direct interaction with PTBP1.

### USP8 promoted the ubiquitination and the degradation of TAK1

**Fig. 6:**
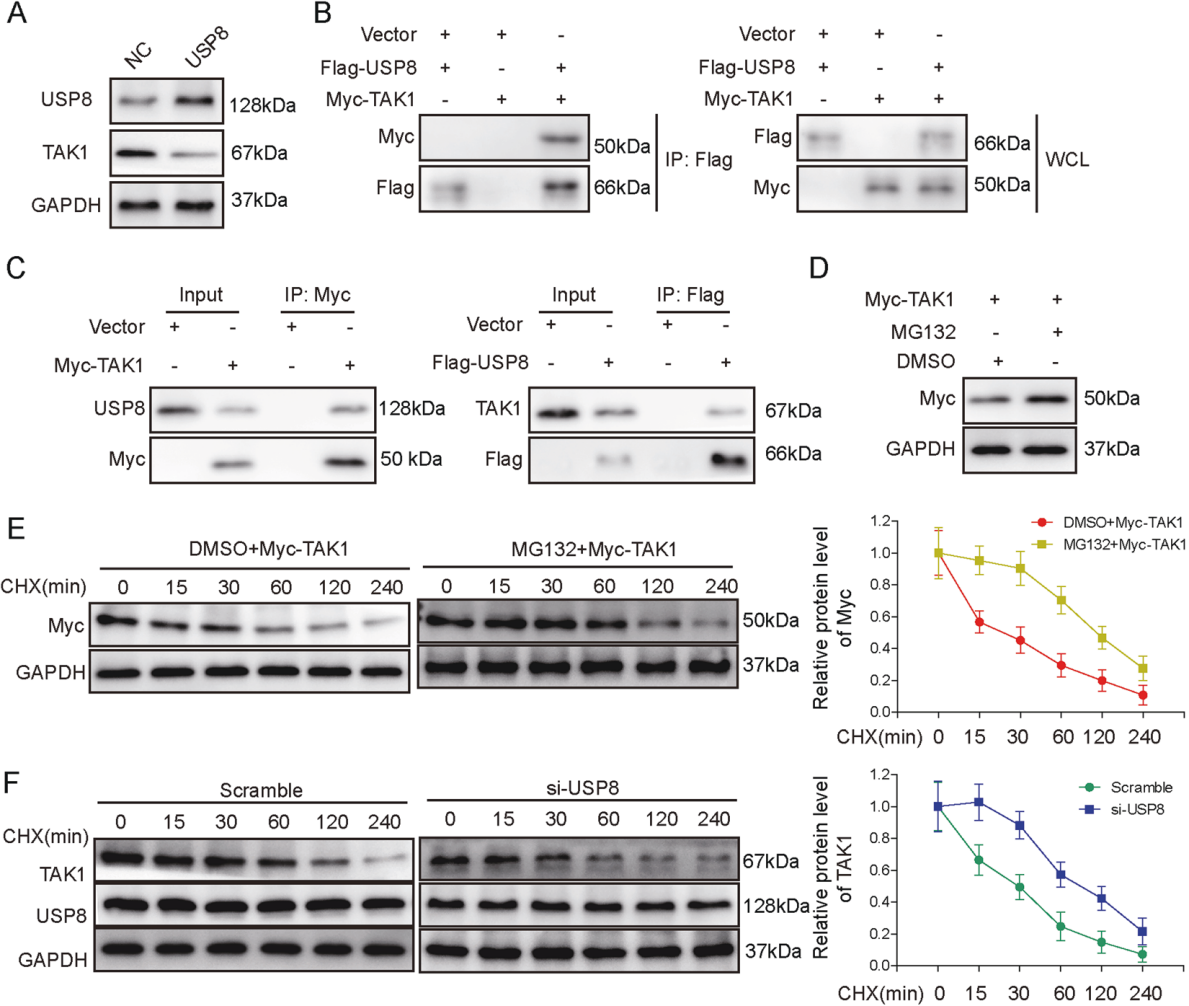
USP8 promoted ubiquitination and degradation of TAK1. **A** The protein levels of USP8 and TAK1 were examined by western blot in KCs overexpressing control (NC) or USP8. **B** The interaction between USP8 and TAK1 was examined by Co-IP in HEK293T cells co-transfected with Myc-tagged TAK1 and Flag-tagged USP8 (left and middle panels). **C** The interaction between endogenous USP8 and Myc-TAK1, and that between endogenous TAK1 and Flag-USP8 was examined by Co-IP in HEK293T cells. **D** KCs were transfected with Myc-TAK1 and treated with or without MG132. The level of TAK1 was examined by western blot. **E** KCs cells from **D** were treated with cycloheximide (CHX) for indicated time periods. The expression of TAK1 was examined by western blot. **F** si-NC- or si-USP8-transfected KCs were treated with CHX for indicated time periods. The protein levels of TAK1 and USP8 were examined by western blot.

Considering that USP8 is a ubiquitin-specific peptidase, we explore the opposite regulation between USP8 and TAK1 by focusing on the ubiquitination of TAK1. First, we overexpressed USP8 in KCs and found that it significantly reduced the expression of TAK1 (Fig. 6A). Upon overexpressing Myc-tagged TAK1 and Flag-tagged USP8 in HEK293T, we could detect their interaction using co-immunoprecipitation (Co-IP) assay (Fig. 6B). In addition, Co-IP also revealed the interaction between Myc-tagged TAK1 and endogenous USP8, as well as that between Flag-tagged USP8 and endogenous TAK1 in HEK293T (Fig. 6C). Furthermore, when protein degradation was blocked in KCs with MG132, ectopically expressed Myc-TAK1 level was increased (Fig. 6D), and its degradation following cycloheximide treatment (to block de novo protein synthesis) was significantly delayed (Fig. 6E), suggesting protein degradation significantly regulated TAK1 level in the cells. A similar delay in the degradation of Myc-TAK1 was also observed in KCs transfected with si-USP8 (Fig. 6F). These data imply that USP8 promoted the degradation of TAK1 cells.

### TAK1 and USP8 oppositely regulated inflammation and pyroptosis in activated macrophages

**Fig. 7:**
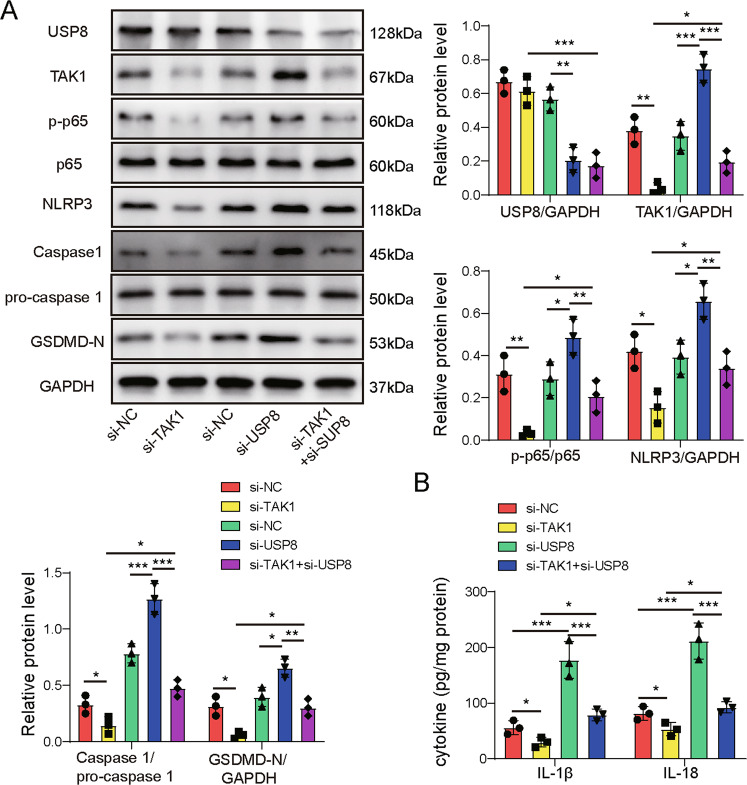
TAK1 and USP8 oppositely regulated inflammation and pyroptosis in activated macrophages. KCs were transfected with si-NC, si-USP8, si-TAK1, or si-TAK1 + si-USP8 and treated with LPS. **A** The proteins levels of indicated targets were examined by western blot. **B** The productions of IL-1β and IL-18 in supernatants of indicated cells were measured by ELISA. *P < 0.05, **P < 0.01, ***P < 0.001.

Lastly, we assessed the significance of TAK1 and USP8 in regulating inflammation and pyroptosis in activated macrophages. Upon transfecting KCs with si-NC, si-TAK1, si-USP8, or si-TAK1 + si-USP8 and treating them with LPS, we found that compared to si-NC cells, si-TAK1 significantly reduced, si-USP8 potently increased, and si-TAK1 + si-USP8 failed to alter the levels of TAK1, p-p65, NLRP3, Caspase-1, and GSDMD-N. In addition, si-TAK1 did not change the level of USP8, although si-USP8 markedly enhanced TAK1 level (Fig. 7A). The same pattern of regulation was also observed for the production of IL-1β and IL-18 (Fig. 7B), suggesting that TAK1 acts downstream of USP8 and they oppositely regulate inflammation and pyroptosis in activated M1 macrophages.

## Discussion

Hepatic macrophages, by producing pro-inflammatory cytokines and activate HSCs, play a critical role in initiating liver fibrosis [7, 17]. In this study, we reveal a novel signaling cascade, METTL3/MALAT1/PTBP1/USP8/TAK1, which involves a wide range of intracellular molecules from an enzyme regulating epigenetic modification, a lncRNA, an RNA-binding ribonucleoprotein, a ubiquitin hydroxyl-terminal hydrolase, to a kinase. Using KCs isolated from fibrotic liver and LPS- or IFNγ-challenged macrophages as our model systems, we showed for the first time that this signaling cascade was not only activated in hepatic macrophages following the progression of liver fibrosis, but essential for inducing pyroptosis and M1 polarization of hepatic macrophages. This study demonstrates how regulations on the epigenetic, transcriptional, post-transcriptional, and post-translational levels cooperatively and collectively link pyroptosis with M1 polarization of macrophages, and further impact liver fibrosis.

As a crucial player in maintaining tissue homeostasis and regulating immune responses, the capability of macrophages to timely alter their activation and polarization status in response to environmental stimuli is important for their heterogeneity and function [18]. Epigenetic modifications, including those on histones, DNAs, and non-coding RNAs, have demonstrated their potency in controlling macrophage activation, polarization, and functions. For instance, histone deacetylase inhibitor, Scriptaid, via activating the expression of glycogen synthase kinase 3 beta, induced M2 polarization of macrophages/microglia and protected against inflammation [19]. The promoter DNA methylation of peroxisome proliferator-activated receptor γ1 was increased by inflammatory cytokine such as TNFα and reduced by TH2 cytokine such as IL-2, which led to the activated M1 and M2 macrophages in adipose tissue, and the aggravated and alleviated insulin resistance in obesity [20]. In this study, we reported not only the relevance of METTL3 (a protein, when dimerized with METTL14, responsible for m^6^A methylation of RNA molecules [21]) to the pathogenesis of liver fibrosis, but its essential role in controlling the stability of lncRNA MALAT1. First, we detected the progressive up-regulations of METTL3 and MALAT1 in KCs from in vivo CCl4-induced liver fibrosis and from in vitro M1-polarized macrophages. These findings were consistent with those reported by Liu et al. which showed that METTL3, but not METTL14, was specifically up-regulated by M1 signals and essentially controlled M1 polarization of macrophages [8]. Second, we identified two potential m6A methylation sites using bioinformatic tools. Using m^6^A RNA immunoprecipitation assays, we showed that both sites were required for m6A methylation and up-regulation of MALAT1 by METTL3. Similar to our data, Jin et al. reported that METTL3-induced m6A methylation and stabilization of MALAT1 contributed to drug resistance and metastasis of non-small-cell lung cancer [10]. In addition, several other studies demonstrated the significance of METTL3 in different hepatic pathologies. Chen et al. showed that METTL3 promoted liver cancer progression via m6A methylation-induced degradation of suppressor of cytokine signaling 2 [22]. Xie et al. showed that METTL3 promotes hepatic insulin resistance via m6A modification-induced stabilization of fatty acid synthase [23]. Lin et al. showed that METTL3 promoted m6A methylation in the CDS and mRNA translation of Snail, contributing to epithelial-mesenchymal transition of liver cancer [24]. Considering that the development of liver fibrosis involves multiple cell types in addition to macrophages, future studies should extend to other cell types and explore the importance and mechanisms of METTL3 in each cell type as well as in liver as a whole.

Mounting evidence demonstrates the value of lncRNAs in disease pathogenesis [25]. When it comes liver fibrosis, several studies reveal multiple pro-fibrotic mechanisms of MALAT1 in HSCs. Yu et al. showed that MALAT1 acted as an endogenous competing RNA (ceRNA) for miR-10b and up-regulated Rac1 expressing, promoting HSC activation [26]. Leti et al. showed that by up-regulating CXCL5, MALAT1 activated HSCs [27]. Studies on MALAT1 in macrophages, however, have not provided a unified conclusion, with some suggesting that it promotes M1 polarization [9, 28] while others showing that it favors M2 polarization [29, 30]. Here we showed that MALAT1 expression was increased in macrophages isolated from in vivo CCl4-induced liver fibrosis or those treated in vitro with M1 stimuli, LPS or IFNγ, which was associated with the up-regulations of multiple pro-inflammatory cytokines and the activation of NLRP3 inflammasome-induced pyroptosis, supporting the involvement of MALAT1 in M1 polarization and pyroptosis of macrophages, as well as the link between these two processes. More importantly, knocking down MALAT1 inhibited M1 polarization and pyroptosis of macrophages both in vivo and in vitro. Similar to our findings, Leti et al. reported that MALAT1 was critical for maintaining pyroptosis of macrophages in diabetes atherosclerosis [27]. However, Leti’s study failed to reveal the mechanism linking MALAT1 to pyroptosis. In this study, we showed that MALAT1, through the direct interaction with PTBP1, promoted the degradation of USP8 mRNA and inhibited USP8-mediated regulation of TAK1 protein.

As an RNA-binding protein and a splicing factor, PTBP1 may activate or repress the transcription of a target gene, by regulating pre-mRNA splicing, polyadenylation, mRNA stability, and/or translation initiation [31]. Studies showed that PTBP1 preferentially activates a target gene when binding to the upstream or within CDS and represses it when binding to downstream sequences [32]. PTBP1, as well as several other RNA-binding proteins, contains a K homology (KH) domain. This domain is required for the interaction with a target RNA molecule containing m^6^A modification [33]. In this study, we detected more abundant binding of PTBP1 to CDS than to 3′-UTR of USP8 mRNA, which was associated with reduced stability of USP8 mRNA. Furthermore, we showed that the binding of PTBP1 to USP8 was significantly promoted by MALAT1. This is the first study, to our knowledge, demonstrating the link and regulation from MALAT1, through PTBP1 and to USP8.

TAK1 essentially activates NF-κB through the phosphorylation of downstream IKK and regulates immune responses [34]. Consistently, multiple studies suggested the importance TAK1 in pro-inflammatory responses of macrophages38-41. TAK1 is also a master regulator for NLRP3 inflammsome-dependent pyroptosis [35]. In this study, we showed that targeting TAK1 was sufficient to abolish LPS-induced activations of NF-κB, NLRP3 inflammasome, pyroptosis, or M1 polarization of macrophages, demonstrating its critical role in controlling these phenotypes. The activity of TAK1, is controlled by ubiquitination, where poly-ubiquitination activates TAK1 [36]. An earlier study by Zhang et al. showed that USP8, through deubiquitination and inactivation of TAK1, suppressed intermittent hypoxia/reoxygenation-induced inflammation in renal tubular epithelial cells [16]. Here we showed similar results. Overexpressing USP8 was sufficient to decrease TAK1 level. Mechanistically, the decrease was associated with direct interaction between these two molecules, and reduced ubiquitination and protein stability of TAK1. Although this study suggests that TAK1 promotes M1 polarization of macrophages and thus may contribute to liver fibrosis, other studies showed that disrupting TAK1 in hepatocytes promoted liver fibrosis and carcinogenesis [37, 38]. Therefore, it is important to assess the impact of TAK1 in different cellular compartments and how they collectively regulate liver fibrosis.

In conclusion, here we showed that the METTL3/MALAT1/PTBP1/USP8/TAK1 axis was activated in macrophages during the development of liver fibrosis. In return, this axis essentially controlled pyroptosis and M1 polarization of hepatic macrophages and aggravated liver fibrosis. This study not only demonstrates alterations from different levels sequentially and collectively regulating biological processes, but also suggests that targeting this axis may benefit the therapy of liver fibrosis.

## Materials and methods

### Carbon tetrachloride (CCl_4_)-induced liver fibrosis model

All animal protocols were approved by the Animal Care and Use Committee of the Xiangyang Hospital No. 2 of Central South University (Changsha, China). C57/BL6 male mice between six and eight weeks of age were purchased from Shanghai Laboratory Animal Research Center (Shanghai, China). CCl_4_-induced liver fibrosis model was established by intraperitoneal injection of 6% of CCl4 (Sinopharm, Beijing, China) prepared in peanut oil at 2 mL/kg bodyweight three times a week for a total of eight weeks, as described previously [39]. Each experimental group included 10 mice. To knock down MALAT1 in vivo, shMALAT1 or negative control shRNA (GenePharma, Shanghai, China) was injected through the tail vein into the mice.

### Isolation of Kupffer cells (KCs) and bone marrow-derived macrophages (BMMs), cell culture, treatments, and transfection

To isolate KCs, livers were excised, minced into small pieces, and digested in RPMI-1640 medium (Gibco, Carlsbad, CA, USA) containing 0.1% type IV collagenase (Sigma, St. Louis, MO, USA) at 37 °C for 30 min. Upon filtering the liver homogenate through a 70-µm cell strainer (Corning, Lowell, MA, USA), KCs were purified using mouse anti-F4/80 microbeads (Miltenyi, Bergisch Gladbach, Germany) according to the manufacturer’s instructions. Isolation and culture of BMMs were performed as previously described [40].

HEK293T and mouse macrophage cell line RAW264.7 were ordered from ATCC (Manassas, VA, USA) and maintained in DMEM medium supplemented with 10% fetal bovine serum and 1% penicillin/streptomycin (all from Gibco) at 37 °C in a moisturized environment containing 5% CO_2_.

To activate and induce M1 polarization of macrophages in vitro, macrophages were treated with 20 ng/ml interferon γ (INF-γ) or 100 ng/ml lipopolysaccharide (LPS; both from Peprotech, Rocky Hill, NJ, USA) for 4 h.

To overexpress METTL3, MALAT1, PTBP1, or USP8, corresponding coding sequences were cloned into pcDNA3.1 vector (Invitrogen, Carlsbad, CA, USA). Empty pcDNA3.1 vector was used as the negative control. SiRNA specifically targeting METTL3 (si-METTL3), si-MALAT1, si-PTBP1, si-USP8, and the corresponding non-targeting control siRNA (si-NC) were purchased from Sigma. Transfections of gene-expressing vectors or siRNAs were performed using Lipofectamine 3000 (Invitrogen) following the manufacturer’s instructions.

### Immunohistochemistry

Mouse tissues were fixed in 10% formalin and embedded in paraffin before prepared into 4-µm sections. Upon deparaffinization in xylene and rehydration through a series of diluted alcohol, tissue sections underwent antigen retrieval in boiled 10 mM citrate buffer (pH 6.0) for 10 min. After blocking endogenous peroxidase activity with 0.3% H_2_O_2_ in PBS for 10 min and non-specific binding with 5% normal goat serum for 1 h at room temperature, the tissue sections were incubated with either anti-F4/80 (ab111101, Abcam, Cambridge, MA, USA) or anti-Ly6c (ab15627, Abcam) antibody at 4 °C overnight. Following the incubation with biotinylated secondary antibody at room temperature for 30 min, the target signal was amplified using Vectastain ABC-HRP solution (Vector Labs, Burlingame, CA, USA) according to the manufacturer’s instructions and detected using Diaminobenzidine (DAB) substrate (Vector Labs).

### ELISA

Levels of IL-1β and IL-18 from mouse liver tissue extracts or cultured supernatants of target cells were measured using ELISA kits for mouse IL-1β and IL-18, respectively (Abcam) according to the manufacturer’s protocols.

### Immunofluorescence

Target cells were cultured on glass coverslips and treated as indicated. Upon fixation/permeabilization with cold 100% methanol for 5 min at room temperature, cells were blocked in TBST buffer containing 2% BSA (Sigma) for 1 h at room temperature and incubated with anti-F4/80 antibody (ab6640, Abcam) diluted 1:200 in blocking solution at 4 °C overnight. After three washes in PBS, cells were incubated with PE-conjugated secondary antibody for 2 h at room temperature. Coverslips were washed four times with PBS and mounted on SuperFrost Plus slides with Vectashield anti-fade mounting medium with DAPI (Vector Labs). Cells were imaged under an Axio Imager A1 microscope (Carl Zeiss, Oberkochen, Germany).

### m^6^A RNA immunoprecipitation

PolyA+ RNA was isolated from KCs transfected with either WT or mutated MALAT1 together with or without METTL3 vs. control or siMETTL3 vs. siNC, and incubated with protein G Dynabeads (Thermo Fisher Scientific) conjugated with anti-N6-methyladenosine monoclonal antibody (ab208577; Abcam) at 4 °C overnight. Mouse IgG was used the negative control. After three washes, m6A RNA was eluted from the beads with 125 μl of 2.5 mg/ml N6-methyladenosine-5′-monophosphate sodium salt (CHEM-IMPEX INT’L INC., Wood Dale, IL) and detected by real-time PCR (RT-PCR), as described previously [10].

### RNA pull-down assays

RNA pull-down assays were performed as described previously19. Briefly, biotin-labeled MALAT1 transcripts and USP8 transcripts corresponding to the coding sequence (CDS) or 3’-untranslated region (3′-UTR) were synthesized using T7 RNA polymerase (Roche, Switzerland), treated with RNase-free DNase I (Roche, USA) and purified with the RNeasy Mini Kit (Qiagen, USA). Biotinylated RNAs were then incubated with HEK293 or KC cell lysates. Streptavidin-conjugated magnetic beads were added, and eluted proteins were detected by western blot analysis.

### RNA immunoprecipitation assay

RIP assay was performed using Magna RIPTM RNA-Binding Protein Immunoprecipitation Kit (Millipore) according to the manufacturer’s instructions. Briefly, cells were collected and lysed in complete RIPA buffer containing a protease inhibitor cocktail and RNase inhibitor. Next, the cell lysates were incubated with RIP buffer containing magnetic beads conjugated with anti-PTBP1 antibody (ab133734; Abcam) or control normal rabbit IgG. The samples were digested with proteinase K and purified RNA was analyzed by RT-PCR to detect MALAT1.

### Co-immunoprecipitation

Myc-tagged TAK1 and Flag-tagged USP8 were constructed using the pcDNA 3.1 vector (Invitrogen) and co-transfected into HEK293 or KC cells. Whole cell lysate was prepared in Co-IP buffer containing 20 mM Tris HCl pH 8, 137 mM NaCl, 1% Nonidet P-40, 2 mM EDTA, and 1×protease/phosphatase inhibitor cocktail (Cell Signaling, Danvers, MA, USA). Total protein (500 μg) was incubated with 1 μg anti-Myc antibody (ab9106, Abcam) or anti-Flag antibody (ab205606, Abcam), or rabbit IgG at 4 °C for 1 h. Then protein A Sepharose beads (Abcam) were added to the mixture and shaken at 4 °C overnight. After washing the beads and associated protein complexes three times, they were boiled in 5× sample loading buffer for 5 min and the supernatant was examined using Western blot.

### Quantitative real-time PCR

Total RNA was extracted from mouse tissues or cells using Trizol reagent (Takara, Dalian, China) and cDNA synthesized using reverse transcriptase (Takara, Dalian, China). RT-PCR was performed with SYBR Green master mix (Takara) following the manufacturer’s instructions with the following primers targeting the corresponding mouse genes: MALAT1 forward 5′-CATGGCGGAATTGCTGGTA-3′ and reverse 5′- CGTGCCAACAGCATAGCAGTA-3′; METTL3 forward 5′-CAGTGCTACAGGATGACGGCTT-3′ and reverse 5′- CCGTCCTAATGATGCGCTGCAG-3′; Ly6c forward 5′-GCAGTGCTACGAGTGCTATGG-3′ and reverse 5′-ACTGACGGGTCTTTAGTTTCCTT-3′; IL-1β forward 5′-GTCGCTCAGGGTCACAAGAA-3′ and reverse 5′-GTG-CTGCCTAATGTCCCCTT-3′; iNOS forward 5′-CAGGGCCACCTCTACATTTG-3′ and reverse 5′- TGCCCCATAGGAAAAGACTG −3′; MCP-1 forward 5′-GTTAACGCCCCACTCACCTG-3′ and reverse 5′-GGGCCGGGGTATGTAACTCA-3′; F4/80 forward 5′-TGACTCACCTTGTGGTC-CTAA-3′ and reverse 5′-CTTCCCAGAATCCAGTCTTTCC-3′; IL-6 forward 5′-AGTTGCCTTCTTGGGACTGA-3′ and reverse 5′-TCCACGATTTCCCAGAGAAC-3′; TNF-α forward 5′-CATCTTCTCAAAATTCGAGTGACAA-3′ and reverse 5′-TGGGAGTAGACAAGGTACAACCC-3′; PTBP1 forward 5′-CACCGCTTCAAGAAACCAGGCT-3′ and reverse 5′-GTTGCTGGAGAAGAGGCTCTTG-3’; USP8 forward 5′- GCCTGCTACAAAGAGTGTTCCAC-3′ and reverse 5’ -GGAGAGGGAAATACTGGCTTGG-3′; and GAPDH (internal control mRNA) forward 5′-GGCATGGACTGTGGTCATGAG-3′ and reverse 5′-TGCACCACCAA-CTGCTTAGC-3′.

### Western blot

The following antibodies were used for western blot (all from Abcam): F4/80 (ab6640; 1:1000), Ly6c (ab77766; 1:1000), NLRP3 (ab263899; 1:1000), Caspase-1 (ab138483; 1:1000), pro-Caspase-1(ab179515; 1:1000), GSDMD-N (ab215203; 1:1000), PTBP1 (ab133734; 1:1000), USP8 (ab228572; 1:1000), TAK1 (ab109526; 1:1000), p65 (ab16502; 1:1000), phospho-p65 (ab86299; 1:1000), and GAPDH (ab8245; 1:5000).

### Statistical analysis

Data were expressed as mean ± SD from three independent in vitro experiments or from all mice from each in vivo experimental group. All statistical analyses were performed with the SPSS 22.0 (IBM, Armonk, NY, USA). Differences between experimental groups were assessed by the Student’s *t* test (for two groups) or one-way ANOVA (for more than two groups) followed by post hoc comparison. A *P* value of < 0.05 was considered statistically significant.

## Supplementary information

Author Contribution Form

## Data Availability

The datasets used or analyzed during the current study are available from the corresponding author on reasonable request.
