# Supplementary figures and images for: The METTL3/MALAT1/PTBP1/USP8/TAK1 axis promotes pyroptosis and M1 polarization of macrophages and contributes to liver fibrosis
Source: Cell Death Discov. 2021 Nov 27;7:368. doi: 10.1038/s41420-021-00756-x (PMC8627510; doi:10.1038/s41420-021-00756-x)

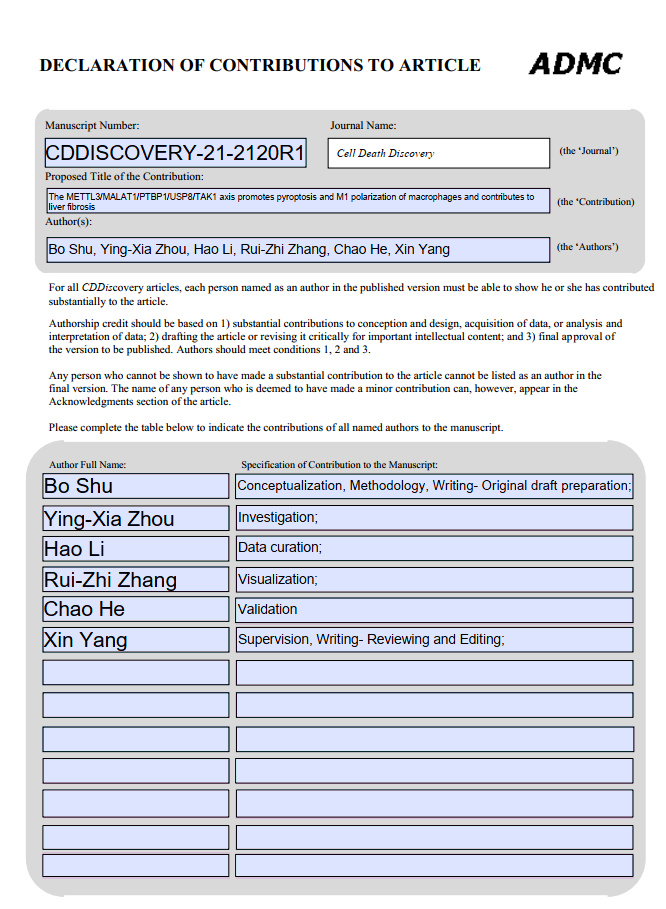

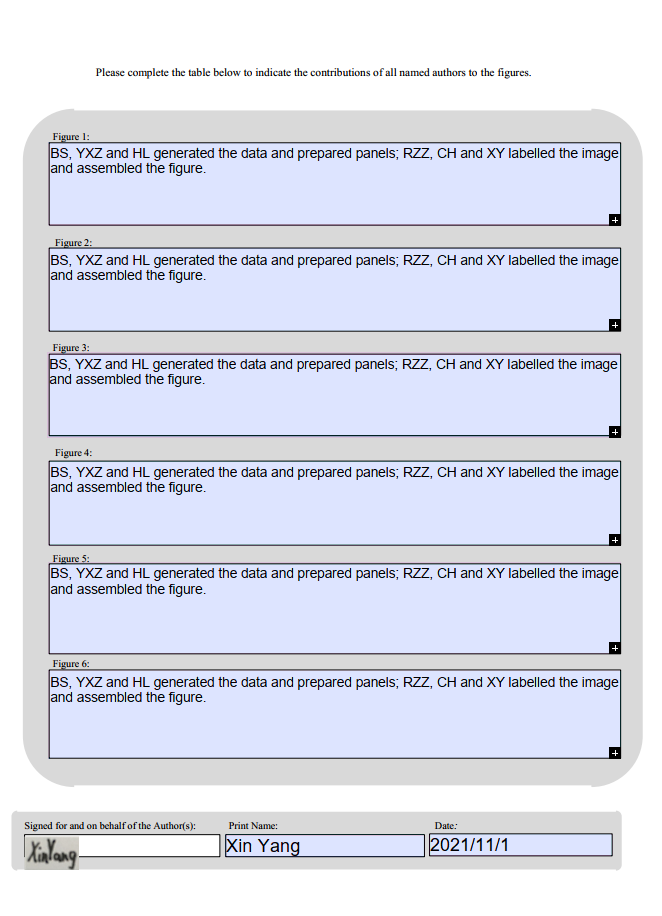

Supplement: Supplementary file 1 — Author Contribution Form [file 41420_2021_756_MOESM1_ESM.docx]
